# Student and staff perceptions of alcohol as part of student life in Denmark: A Q methodology study

**DOI:** 10.1371/journal.pone.0205923

**Published:** 2018-10-25

**Authors:** Stine Holmegaard Christensen, Bridgette M. Bewick, Louise Bryant, Mette Vinther Skriver, Mette Terp Høybye

**Affiliations:** 1 Interdisciplinary Research Unit, Elective Surgery Centre, Silkeborg Regional Hospital, Silkeborg, Denmark; 2 Division of Psychological and Social Medicine, School of Medicine, University of Leeds, Leeds, United Kingdom; 3 Section of Health Services Research, Department of Public Health, Aarhus University, Aarhus, Denmark; 4 Interacting Minds Centre, Department of Clinical Medicine, Aarhus University, Aarhus, Denmark; Northumbria University, UNITED KINGDOM

## Abstract

**Introduction:**

Intervening effectively to prevent students’ harmful use of alcohol remains a challenge. Harmful alcohol use has been noted as the most dominant public health problem facing universities today. This study sought to investigate the diversity in staff and student perceptions of the contribution alcohol makes to student life in a Danish university setting. Increasing understanding of staff and students’ perceptions of how alcohol fits into student life is required to amend future public health intervention for this population.

**Materials and methods:**

This Q methodology study included 38 staff members and 105 students from Aarhus University, Denmark. Participants used online Q sorting software, to rank 40 statements about the contribution alcohol makes to the university student experience from strongly agree to disagree. To support the interpretation of the factors, self-reported alcohol consumption and demographic data were collected. In addition qualitative data was collected on the participant’s reasons for the ranking of the items they most strongly agreed or disagreed with.

**Results and discussion:**

Using principal components analysis, five statistically independent viewpoints for students and four for staff were identified.

The findings provide evidence to inform approaches to prevent harmful alcohol use. Some viewpoints suggest a need for tailored secondary and tertiary prevention and intervention that focusses on individuals and/or sub-groups who are at risk of consuming alcohol at harmful levels. Other viewpoints suggest the need for primary universal prevention to support the maintenance of healthy norms which can prevent harmful alcohol behaviour. Public health campaigns need to ensure that interventions targeting harmful alcohol use at universities challenge problematic perceptions and attitudes while also bolstering exposure to positive norms.

## Introduction

A large consumption of alcohol has a harmful effect on health. According to WHO harmful alcohol use is one of the world’s leading risk factors for morbidity, disability and mortality [1]. Europe is the region of the world with the highest per capita alcohol consumption. Students’ drunkenness is more common today compared to earlier generations and international surveys show an association between the level of education and alcohol patterns of the youth [2,3]. Students attending university, on average, consume more alcohol than their non-student peers, and harmful alcohol use is the most dominant public health problem facing universities [2–7]. From a public health perspective, knowledge is needed on how to effectively intervene to reduce students’ harmful alcohol use. Candidates from the education programs of medicine, public health science and dentistry will go on to play a key role in public health strategies of early alcohol detection and prevention of alcohol related diseases. If their harmful alcohol behaviour is continued after graduation, their individual alcohol perceptions and behaviour could potentially impact their professional practice [8–9].

Human behaviour and health is highly influenced by societal expectations and the social norms and alcohol behaviour is no exception [10]. In a university setting social norms are not solely determined by student peer interactions but are also influenced by university staff views on student alcohol behaviour [11]. The social norm is also influenced by the structural norms of the university policies on alcohol. Existing literature on interventions targeting problematic health behaviour suggest, we need to understand the diverse perceptions and views on alcohol consumption to develop useful interventions in the target population [12]. This study has an aim of investigating the diversity in staff and student perceptions of the contribution alcohol makes to student life in a Danish university setting.

## Materials and methods

This study was approved by the Danish Data Protection Agency (1-16-02-522-16). Furthermore, informed consent was gained from all participants. Relevant data of the participants were allowed published, but only as anonymous and not traceable data.

Ethical approval for the study was granted by the University of Leeds School of Medicine Research Ethics Committee (approval number: SoMREC/14/052). In accordance with Danish research ethical regulations, further ethical approval was not required.

### Q methodology

Fusing aspects of both quantitative and qualitative research, Q methodology allows the range of subjective perspectives on a topic to be observable to others [13–15]. A Q methodological study involves several steps [16]. First, a sampling of statements from the population of things ‘written or said’ about the topic, termed the concourse [17]. From this concourse, through a series of iterative steps, a sample of statements that represent the concourse are is identified; this sample is termed the Q-set. The respondents for the study are selected to achieve diversity of viewpoint on the topic rather than statistical representation of the target population. Respondents are required to rank the Q-set along a continuum of preferences, for example, from strongly agree to strongly disagree; this is termed Q-sorting. The resulting Q-sorts are taken to represent the viewpoint of that individual. All Q-sorts are then subjected to data reduction using factor analytic techniques, to identify viewpoint ‘types’—each independent factor being taken to represent an independent viewpoint in social discourse. In Q methodology the analysis correlates participants. instead of items, in order to identify groups of participants who share a similar viewpoint. Interpretation of these factors using statistical information is usually supported by some qualitative data, for example from interviews or open-ended questionnaire type responses. These interpretations produce a narrative description of each of the viewpoints [13,15].

### Sampling the concourse and creating the Q-set for this study

The concourse of this study could be defined as the socially available discourse (things written or said) about student alcohol drinking as part of University life [18]. The concourse was sampled and a Q-set developed during a previous study by Yule et al at University of Leeds [18]. The Q-set created by Yule et al had been generated through a wide-ranging review of a range of sources: (A) research articles and unpublished academic material describing attitudes and behaviors of students in relation to alcohol and student life, (B) online newspaper and magazine articles about student drinking (C) students’ responses to these online articles, and (D) relevant websites, articles, blogs, YouTube clips and documentaries [18]. Forty statements from the concourse were selected by Yule et al. as representative of the full sample, and the final Q-set for use in the questionnaire was created and pilot tested at University of Leeds, S1 Appendix [18]. The statements were translated from English into Danish through a consultative process of back-translation done by the members of the research team at the host university and a communication and language consultant at Silkeborg Regional Hospital. To ensure that the translation of the items captured the original meaning and were relevant to Danish culture there was a continuous dialogue between the British and the Danish research team and all changes to wording were logged and mutually agreed, S1 Appendix. The Q set items were entered into the online Q sorting platform (POETQ) used for data collection [19].

### Additional data: Background characteristics of the sample and assessment of alcohol consumption

To obtain background data on our sample a short questionnaire was added to the online Q-sorting platform. Students were asked about their age, gender, affiliated department and current student status. Staff were asked about their age, gender, associated department, description of employment and their amount of contact with students. This was done to assess diversity of participants and to understand whether specific viewpoints are associated with specific characteristics [15]. Drinking behavior of each participant was assessed using the validated AUDIT measure (Alcohol Use Disorder Identification Test), S1 Appendix [20]. The AUDIT screening questions, regarding recent alcohol use, alcohol dependence symptoms, and alcohol-related problems were responded using a score (0–4) for each item with a possible maximum score of 40 [20]. A total score of 0–7 defines an individual as having a little risk of alcohol problems, 8–15 defines a middle risk, 16–19 classifies an individual as having a high level of alcohol problems, and a score of 20–40 defines individuals within a risk of possible dependency on alcohol [20]. Both the demographics and the AUDIT score was used in the interpretation of the data.

### Study population

Student and staff members from education programs in medicine, public health science and dentistry at Aarhus University, Denmark were invited to participate in the study. Staff members employed within the Department of Clinical Medicine, Biomedicine, Public Health or Dentistry were identified via the University website. All staff in these departments were invited to participate by a personal e-mail send out by the research team in March 2017. Students were approached through Facebook posts for relevant student groups and by placing posters in relevant buildings around the University campus [21]. It was not possible to recruit students directly through personal e-mail due to University policy. Two weeks later a second post was made in the Facebook groups targeting students specifically from public health science and dentistry to increase the number of student participants from these two programs. In addition, 354 of the 942 staff members were randomly selected and sent a reminder about the questionnaire by e-mail. A total of 38 staff and 105 students responded to the invitation to participate.

Table 1 shows the demographic characteristics of staff and student participants and data on self-reported alcohol consumption: Of the student respondents, 55% were enrolled in medicine, 29% were enrolled in public health science and 18% were enrolled in the dentistry program.

**Table 1:** Characteristics of the study participants.

	Student		Staff	
Participants N (%)	105		38	
Age in 2017, ±SD	24	± 2.5	45	± 11.2
Gender				
Female, n (%)	81	77%	29	76%
Male, n (%)	23	22%	9	24%
Transgender, n (%)	1	1%	0	0%
Description of employment, staff				
Scientific staff, n (%)	-	-	29	76%
Technical and administrative staff, n (%)	-	-	9	24%
Contact with students, staff				
Monthly contact	-	-	6	16%
Weekly contact	-	-	12	31%
Daily contact	-	-	17	45%
No contact	-	-	3	8%
Current student status				
Bachelor	43	41%	-	-
Master	61	58%	-	-
Other	1	1%	-	-
Full-time student, n (%)	103	98%	-	-
Consumed alcohol, n (%)	101	96%	33	87%
Average Audit score*, ±SD	9.8	± 5.3	4.9	± 2.6
0–7 points, n (%)	38	38%	33	89%
8–15 points, n (%)	51	51%	4	11%
16–20 points, n (%)	9	9%	0	0%
≥20 points, n (%)	2	2%	0	0%

SD, Standard Deviation

*Individuals missing, N: Students: 10, Staff: 2.

### Data collection and analysis

The online Q-sorting software POETQ was used to collect both quantitative and qualitative data (19). The Q-sort data (quantitative) were analysed using PQMethod version 2.33 [22]. Interpretation of the factors used statistical outputs from PQMethod alongside qualitative data collected as part of the online sorting procedure.

**Fig 1:**
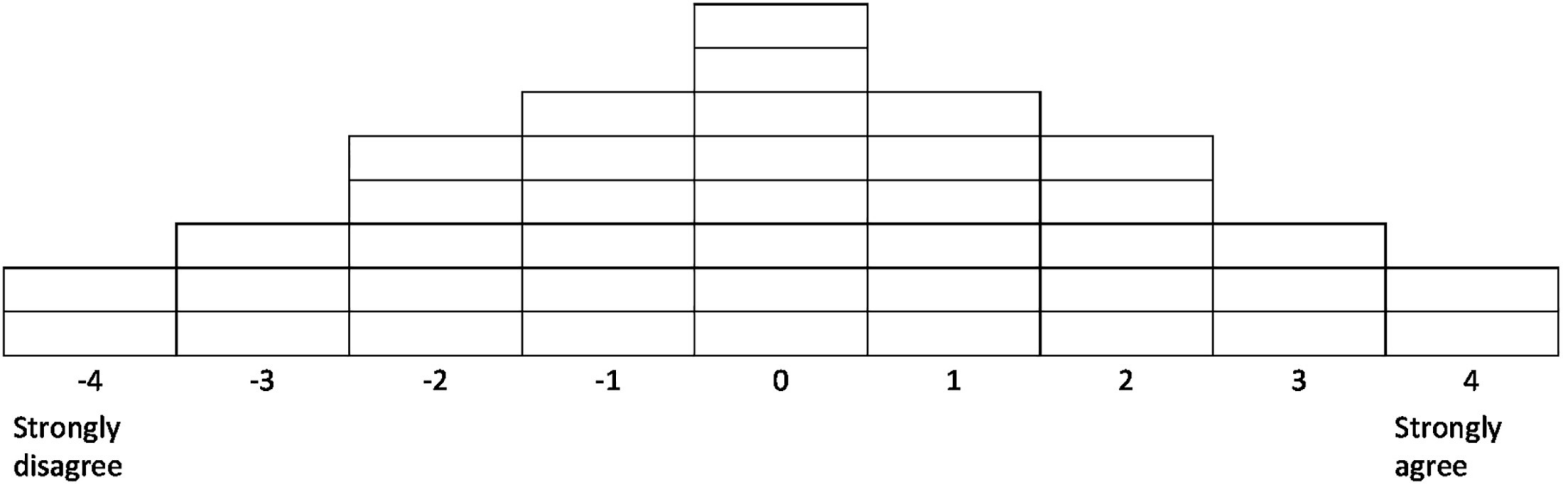
The Q-sorting grid.

Through a series of iterative steps the POETQ online platform enabled participants to systematically rank the 40 statements according to agreement or disagreement, ultimately placing the items into cells on a normally distributed grid, Fig 1 [16,23]. Qualitative data were collected within POETQ: respondents were asked to complete free text entries to give their reasons for selecting the statements they ranked highest and lowest in terms of agreement or disagreement. The Q-sort data were downloaded into an Excel data file. Quantitative data were submitted to statistical analysis using PQMethod. The Q-sorts of staff and student were analysed separately. Qualitative data entered within POETQ were extracted from the Excel file and used to support interpretation of the factors.

### Factor extraction and interpretation

Using PQMethod, factors were identified using Principal Components Analysis (PCA) and Varimax rotation. Established strategies were employed to identify the maximum number of interpretable and distinct viewpoints to extract and take forward for interpretation [16,23]. Firstly, factors with an eigenvalue greater than one (Kaiser-Guttman criterion) with at least two significantly loading Q-sorts were plotted on a simple line graph (scree plot): factors falling around the point where the line changed slope and before the point where the line leveled off were considered for rotation. Secondly, Humphrey’s rule was applied using the cross-product of the two highest loading sorts on each factor [16], Q-sorts which load significantly on only one factor are considered ‘exemplars’ of the factor [16]. The Q methodology software uses a weighted formula to merge the exemplars to create an average score for each of the 40 statements. This is called the factor array and represents an idealized Q-sort for each viewpoint [16].

When considering the factor solution for students, a bipolar factor was identified within the ‘best fit’ four factor solution as both positive and negative significantly loading Q-sorts occurred within one of the factors. By inverting a copy of the bipolar factor a five factor solution was created, which accounted for 60% of the variance. Seventy-three of the 105 (70%) students mapped significantly onto one of the factors. This five factor structure was selected for interpretation. For staff, a four factor solution was identified as most suitable for interpretation: these four factors accounted for 53% of the variance: 25 of the 38 staff (67%) were mapped significantly into one of the factors. The factor arrays for students and staff are shown in Table 2. This five factor structure was selected for interpretation.

**Table 2:** Factor arrays: Scores against each item by viewpoint for student and staff.

No	Statement	F1	F2	F3	F4	F5	F6	F7	F8	F9
1	It is normal for students to use alcohol to relieve stress and worry	2	-1	-2	-1	0	-3	-3	-1	-3
2	It can be expected that students will feel pressure from their peers to drink alcohol	1	-1	0	2	2	-1	-1	2	3
3	Drinking alcohol at home before a night out is a normal part of student life	3	3	3	2	0	2	4	3	4
4	Being unable to remember parts of a night out due to drinking alcohol is an expected part of the student experience	-4	-2	-1	-2	-3	-1	-3	-3	-2
5	Students are more interested in partying and drinking alcohol than studying	-2	-4	-4	-3	1	-4	-4	-2	-4
6	After a student night out drinking alcohol it is accepted that there may be regrets the next day	-1	2	1	-2	-1	1	1	-1	-1
8	The way students behave when drunk is disgusting	0	-3	-3	0	4	0	-3	0	-1
9	Most students who prefer not to drink find it is easy to avoid alcohol	0	1	-1	-4	0	2	0	-2	-1
10	Behaving like an idiot when drunk is seen as a normal student behaviour	-3	-4	-2	-1	-2	-3	-4	-1	-2
11	Students who are drunk don’t care about the consequences of their actions on others	0	-1	-3	0	3	0	-2	2	1
12	A lot of student conversations revolve around alcohol and drinking	-2	-3	0	1	-1	-4	0	1	0
13	It is expected that academic work will be affected at some point due to the consequences of drinking alcohol	-1	1	-1	0	1	3	-2	2	1
14	A good student party needs alcohol	-2	1	1	-2	-4	1	3	-3	0
15	Students that drink a lot of alcohol are often viewed as the most popular	1	-1	2	2	0	-2	-1	-2	-2
16	Students view feeling unwell the next day after drinking too much alcohol as a sign of a good night out	-1	-1	0	0	-1	0	0	0	0
17	Students that don’t drink alcohol are often viewed as boring	1	-2	1	4	1	-2	1	2	-1
18	It is easier for students that drink alcohol to make friends	1	0	2	3	-1	-2	2	-1	0
19	Students live for today; they don’t think about the effects of alcohol on their health	0	2	-2	1	0	1	0	0	4
20	Students enjoy spending a night at home with friends and not drinking, just as much as going out and drinking alcohol	4	4	3	0	2	4	2	1	3
21	It is expected that students can drink a lot of alcohol; it’s not acceptable for students to get drunk on just a few drinks	-4	-3	-2	-1	-2	-3	-2	-2	-3
22	Students get tired of social events that are organised around drinking alcohol	1	-1	-3	-2	3	1	-2	0	-2
23	Students often drink alcohol so that they can fit in with other students	2	0	0	3	0	1	1	3	2
24	Students like the fact that drinking alcohol lowers their inhibitions and enables them to do things they wouldn’t normally do	3	1	1	1	2	0	4	4	0
25	Most students drink sensibly or not at all	2	0	0	-3	-2	3	-1	0	-4
26	The beginning of the academic year is typically all about getting drunk	-3	1	-4	4	4	0	0	3	0
27	Drinking alcohol is a strategy used to gain confidence by many students	0	0	0	1	0	-1	2	4	-2
28	Students think that getting drunk with friends is a good thing	3	3	4	2	1	2	3	1	1
29	An important part of the student experience is being free to drink alcohol	-2	0	1	-1	-3	1	-2	-2	1
30	Being a student is the best time to drink alcohol because students have fewer responsibilities	2	0	2	0	-2	0	2	-1	2
31	Nights out with friends getting drunk provide some of the best memories of student life	-3	2	4	-1	-3	-1	2	-4	2
32	Students care more about being healthy these days and so the amount of alcohol they drink is reducing	-2	0	0	-3	1	3	0	1	0
33	Students think drinking alcohol blocks out negative emotions	0	-2	-1	-1	-2	-2	1	1	-1
34	Most students are good at knowing when to stop drinking alcohol so that they don’t get too drunk	2	2	-1	-2	0	2	0	0	-1
35	Students find it easy to admit to other students that they do not like drinking alcohol	-1	0	-2	-4	-1	2	-1	-3	-3
36	Students are thoughtful about when to drink alcohol, taking academic obligations such as essays or exams into account	4	4	2	1	2	4	3	0	0
37	Games that involve drinking alcohol are a valued part of the student drinking experience	1	3	3	1	2	0	0	0	1
38	For students who work hard, a night out drinking alcohol and getting drunk is well-deserved	-1	2	2	0	-4	-1	1	-4	1
39	Students spend too much money on alcohol rather than items like food or academic books	0	-2	-1	0	3	0	-1	1	2
40	It is normal to see students finishing their drinks fast and in one go so they get more effect from the alcohol	1	1	1	2	1	-1	2	2	2

Established methods of Q factor interpretation were applied to the factor solutions for the student and staff data in turn. Interpretation requires a careful synthesis of the quantitative and qualitative data collected during the Q-sorting activity. The information produced by PQMethod is used to inform the first level of interpretation. Using the factor arrays, the highest and lowest scores assigned to particular statements for each factor are considered first. Next statements identified as statistically distinguishing for that factor at *p* < 0.01 are considered to identify what is unique about the factor. A deeper level of interpretation then follows whereby the idealized Q-sort is considered alongside the qualitative information provided by the participants, in this study collected via the online platform. The qualitative data is used as a ‘validity check’ against the researcher’s interpretation and to throw more light on the importance of certain statements to this particular viewpoint and the meaning they may have.

## Results

The results are presented as a set of narrative descriptions of the different viewpoints identified via the factor analysis. Qualitative data from participants who were exemplars of each factor are used to illustrate and provide evidence to support the researchers’ interpretation.

### Viewpoint I (students): Students drink sensibly

Viewpoint I was exemplified by five exemplars, seventy-eight percent of whom were women. They had an average age of 24 years and their average AUDIT score was 6.2.

Distinguished from all other viewpoints, students are here perceived to be drinking sensibly. There is a sense that students within this viewpoint use alcohol differently than those in the other viewpoints, as alcohol in this viewpoint does not play a central part in their social interaction with friends or is not central to activities at the start of term. Students do drink with friends, but here drinking games are not important. Having a high alcohol tolerance is also not expected, and students are expected to be able to remember the events of a night out partying. But students do use alcohol to lower their inhibitions and they feel able do things they would otherwise not do.

*“I do not feel like there is pressure to be able to drink a lot of alcohol*. *If you get drunk from a few units it is also “alright”*. *I do not think that others care how much alcohol one is able to consume before they get drunk”*

Participant(P): 25

The best memories of student life are not necessarily perceived as related to alcohol as a night at home with friends without drinking is just as enjoyable to these students as nights out drinking alcohol. Alcohol is therefore not used as a social driver but rather used to relieve stress and concerns, and much less related to partying with friends.

*“Periods with exams*. *Easy way to calm down afterwards”*

P: 2

Students in viewpoint I believe that most students like to party with their friends but when doing so students drink alcohol sensibly. There is a sense that students use alcohol not as a social driver but as a way to relieve stress and concerns.

### Viewpoint II (students): No pressure but alcohol and parties makes the best memories

The 36 exemplars have an average age of 24, and 78% of them are women. Their average AUDIT score was 10.7.

It is believed in this viewpoint that those who do not drink miss out on some of the best memories as a student; Viewpoint II believe that some of the best memories are those of social encounters involving alcohol. Getting drunk plays, a central part at the start of a new semester for students in this viewpoint. There is a sense of recklessness associated with drinking, as drunk students are seen not to care about the health effects of their behaviour.

*“It is just so far into the future*, *and we cannot know the consequences of our [drinking] choices*, *like you for instance do with smoking*. *You just think*:*—this is not going to affect me*, *I am healthy otherwise”*

P: 61

But this does not mean that students are not conscious about how they act when they are drunk.

There is a belief that students put studying above partying and plan their drinking around study activities. These students believe that no one should feel pressured from fellow students to consume alcohol.

*“It is not my perception at all that those of my fellow students who do not drink are perceived as boring*. *A lot of those who do not drink on my education program are still going to the parties”*

P: 78

Particular to this viewpoint is the perception that students are not seen as boring if they choose not to drink alcohol and they acknowledge that nights in with friends and other activities without alcohol are also an important part of a social life.

“Nights in, like movie nights, nights with good food for example, are just as important as night on the town”

P: 13

This viewpoint is distinguished from the others by a belief that students who do not drink are not seen as boring. There is a perception that students do not feel pressured or expected to drink alcohol if they do not want to. Even though drinking is not more important than studying, drinking is still central to the start of term, and nights out partying can be fun and provides some of the best memories of your time as a student.

### Viewpoint III (students): Nights out drinking are the best

Eight exemplars, sixty-tree percent women, with an average age of 23 years are represented within this viewpoint. The highest average AUDIT score of 12.4 is found here.

Students in viewpoint III cherish the memories of nights out partying and getting drunk with friends as some of the best. They do not complain about social events being centered around alcohol. They view alcohol as playing an important role in facilitating social interaction.

*“[I] Have never experienced anybody complaining about too many events around drinking alcohol*. *If you do not want to drink alcohol*, *you can just stay away”*

P: 26

However, these students do not believe getting drunk is what the start of term is all about, and there is a perception that students put their studies before partying and drinking. Weekends spent partying and drinking with friends are seen to be a good way to socialize after a long week of studies.

“Because you often study on your own during the week, it is enjoyable fun when you go out on a Friday night to have fun and party with fellow students”

P: 98

The health risk of a high alcohol intake is not of concern for students in this viewpoint. In contrast to viewpoint I, students in viewpoint III do not believe it is normal to use alcohol to relieve stress and concerns.

According to students in viewpoint III nights out drinking with friends provide some of the best memories of student life. Nevertheless, they believe students still care about the consequences of their drinking behaviour. They believe students prioritize their studies over drinking.

### Viewpoint IV (students): Students drink to fit in

In average the 21 exemplars in this viewpoint are 24 years old. Eighty-six percent of them are women and their average AUDIT score is 8.2.

Students within viewpoint IV are distinguished from all other student viewpoints by the perception that students are expected to drink and that they do not find it easy to avoid alcohol or admit if they do not drink. Viewpoint IV believes this sense of social pressure leads to most students drinking to fit into the group and to avoid being perceived as boring.

*“Not so much to explain here*. *That is the way it is*. *If a person does not drink alcohol the majority will judge a student as boring*. *It is probably because alcohol plays such a central part for the youngsters in Denmark”*

P: 33

For students in viewpoint IV the start of term seems to be about getting drunk, and alcohol consumption is portrayed as the way to make new friends.

*“Alcohol is a huge part of student life*. *Especially in the beginning when fresher’s week is going on*, *all social events include alcohol*. *It is my perception that you are perceived as strange if you do not drink alcohol at these social events*. *You need a good excuse*, *just saying you do not feel like drinking is usually not enough”*

P: 45

Still, they do not believe that nights out partying with friends provide some of the best memories for their time as students, instead they believe the best memories could easily occur somewhere else.

Students from viewpoint IV feel expected to consume alcohol to avoid being classified as boring, and to fit in and make new friends. For these students, such pressure is not seen as a positive and they do not believe that the best memories come from times spent drinking alcohol.

### Viewpoint V (students): Other students are more interested in partying

In this viewpoint three exemplars are found. All are women and with an average age of 23. The average AUDIT score is 2.5 which is the lowest AUDIT score of all the factors.

For the students within this viewpoint, alcohol and drinking excessively is perceived as part of student life. Drunk students are considered repulsive. Drunk students are not seen to worry about their behaviour. There is a perception within this viewpoint that students in general find partying more important than studying. Students who exemplify this viewpoint do not consider alcohol central to a good party and often they themselves do not drink. These students are fed up with social events revolving around alcohol. They do not think alcohol should be a reward to hardworking students, neither do they agree that a night out partying and drinking is well deserved after finishing a period of exams.

“I do not think that it is a reward to get drunk, and I do not drink myself”

P: 31

Viewpoint V holds a different moral perception of alcohol and alcohol behaviour than is seen in the other viewpoints and they are tired of social events where alcohol is central.

“Many start shouting when they are drunk, and many lose their inhibitions”

P: 71

Viewpoint V holds a different moral perception of alcohol and alcohol behaviour than what is seen in the other viewpoints. There is a perception that drunk students are repulsive and that they do not worry about their behaviour. They believe that for other students the beginning of term has a focus on getting drunk, Students in viewpoint V are tired of social events driven by alcohol. There is a belief that most students prioritize partying over their studies, but the exemplars do not believe that a good party calls for alcohol.

### Viewpoint VI (staff): Students drink sensibly

Ten exemplars represent this viewpoint, and in average their age is 48 years. Seventy percent of them are women and sixty percent of the exemplars have daily contact with students. The average AUDIT score is 4.1, and together with viewpoint 8 this is the lowest AUDIT score within the staff viewpoints.

Viewpoint VI is the only staff viewpoint where students are perceived to drink alcohol sensibly. Students are seen to be in control of their drinking, to know when they have had enough, and to avoid downing their drink. Furthermore, they remember what happened on a drunk night. There is no expectation that students are able to drink a lot of alcohol before getting drunk. Staff in viewpoint VI believe that the student population have reduced their alcohol consumption within recent years and have become healthier.

*“I have seen research documenting decreasing alcohol consumption among young people*. *Young people today are also under so much pressure that they must be disciplined and sensible in every way”*

P: 97

Staff do not see alcohol as central to student life, nor do they believe that nights out partying provides some of the best memories. They believe that students plan their drinking around study commitments. Particular to this viewpoint is the perception that students engage in activities that do not include alcohol and that not many conversations between students are centered on drinking and partying.

*“Students are very social and meet up often*, *including during the week*. *They only drink alcohol when they party”*

P: 38

Given this controlled use of alcohol among students those who prefer not to drink find it easy to avoid alcohol and to admit that they do not drink.

Staff in this viewpoint think that students drink sensibly, and they do think that academic work of students is likely to be affected when partying and drinking alcohol.

Staff from viewpoint VI are more likely to think students drink sensibly when compared to other student or staff viewpoints. At the same time, they think that students have reduced their alcohol intake in recent years to take on a healthier lifestyle. Drinking nights with friends are not seen to provide the best memories. Alcohol is not central to student life, and it is easy for students who do not drink to avoid alcohol. It is also interesting that staff from viewpoint VI think that the academic work of students’ is likely to be affected when students in the five viewpoints in general do not.

### Viewpoint VII (staff): Students drink heavily

The five exemplars in this viewpoint have an average age of 41 years; eighty percent of them were women. Eighty percent of the exemplars had monthly or weekly contact with students. Their average AUDIT score was 4.6.

Within viewpoint VII staff perceive that most students consume large amounts of alcohol and enjoy drinking with friends. Preloading before going out drinking is normal for students, and alcohol at parties is seen as necessary for it to be fun. Particular to this viewpoint, staff hold a perception that students use alcohol to make new friends and shared with viewpoint VIII they believe that students use alcohol to gain confidence. There is a sense within this viewpoint that some students drink because they feel pressured to. There is a sense in this staff viewpoint that those students who do not drink are perceived as boring. Drinking alcohol is seen as important for staying within the general social norm.

*“People*, *who do not drink are often told that they are boring and meanwhile they must give a reason why they do not drink*. *Those who drink are "the normal" people*. *Alcohol is often a social thing*. *I find that not drinking alcohol is seen as not wanting the social time together”*

P: 27

No matter why students choose to drink, the staff in this viewpoint believe students’ academic obligations are not affected by it, as study commitments are always prioritized over partying.

*“In my opinion*, *most people in higher education do take their studies seriously*. *Therefore*, *it is not more important to party than to study*. *If that was the case*, *they would not fight the battle it is to finish a university degree*. *Drunkenness and partying is just a way to get away from the pressure and expectations”*

P: 69

According to staff in viewpoint VII, students do consume a lot of alcohol and get drunk. They believe that some students drink for fun and to make new friends while others find it difficult to avoid and drink to ensure that they are not seen as boring. Whatever reason they have, staff in this viewpoint believe students’ study obligations are not affected by drinking alcohol.

### Viewpoint VIII (staff): Students drink alcohol and those who do not drink are perceived as boring

Seven exemplars, in average 48 years old, are in this viewpoint. Eighty-six percent are women and forty-three percent of the exemplars have daily contact with students. Together with viewpoint I, the lowest average AUDIT score (4.1) of all is represented here.

Of all staff viewpoint, staff in viewpoint VIII are the only ones with the perception that the start of term is all about getting drunk. They find that alcohol is used by students as a strategy to lower inhibitions and gain confidence. Drinking is perceived as helping students to not come across as boring to fellow students. At the same time, they agree that students feel some peer pressure to drink alcohol.

“Especially because it is used as a tool to get to know each other”

P: 24

*“Students are often called boring and other things if they do not want to join in and being part of drinking alcohol*. *You need to have a strong and determined personality to avoid it”*

P: 12

While alcohol is seen as pivotal in making new connections this viewpoint does not believe that the best memories are because of consumption of alcohol. Staff represented in viewpoint VIII believe good memories from one’s time as a student may as well be from nights in with friends without alcohol and that alcohol is not necessary to have a good party.

“Things other than alcohol decide whether or not it is a good party”

P: 9

Alcohol is not a reward, and students do not deserve a night out partying and celebrating after exams.

In viewpoint VIII staff believe that for students the start of term is about getting drunk. The perception is that students drink alcohol to gain confidence and to not come across as boring. Alcohol plays a function for students, but it is not seen as central to student life. Alcohol is not found to be needed to have a good party, and it should not be seen as a reward for students celebrating their hard work.

### Viewpoint IX (staff): Student drinking is a problem

The three exemplars have an average age of 36 years. They are the youngest and also those with the highest average AUDIT score (5.3). All exemplars had daily contact with students and two out of three were women.

Within this viewpoint staff hold the perception that students do not drink sensibly but instead that their drinking is a problem. They believe that students do drink before going out partying, and that student drinking is ‘out of control’, as students do not know when to stop.

“Many get very drunk”

P: 35

It is perceived that students do not care about the health effects of drinking and the potential later consequences of their alcohol behaviour is therefore not a concern for students.

Some of this reckless behaviour is seen to be due to a social expectation and peer-pressure among students, where some find it hard to admit if they do not want to drink.

*“I think about when new students start at university*, *and they are going on fresher’s week*. *Then it is expected that they drink”*

P: 6

According to staff in viewpoint IX most students do not drink sensibly, and they do not know when they have had enough. They do not care about the possible health effect of their behaviour. There is also a sense within this viewpoint that staff think that students feel expected to drink such high amounts of alcohol.

### Consensus statements

No consensus statements were found within the viewpoints for students. For staff three consensus statements were found, as exemplars loaded equally by either answering “neutral” or “disagreeing” to statement 15, 16 and 21. This reflected a shared perception that students are not expected to be able to drink a lot of alcohol before getting drunk and that students drinking a lot are not the most popular. Additionally, all viewpoints were neutral towards the statement that students view feeling unwell the following day after heavy drinking to be a sign of a good night out.

#### Comparison of the viewpoints for staff and students

**Fig 2:**
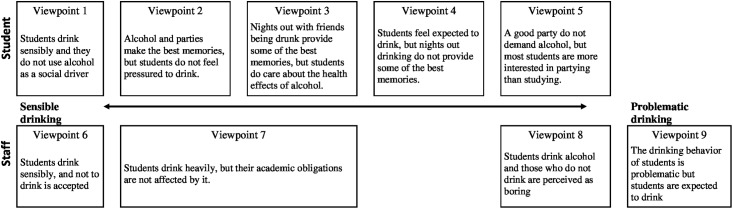
How the viewpoints for staff and students fit together.

This study aimed to explore the diversity in perceptions among staff and students of the contribution alcohol makes to student life. Nine different viewpoints for students and staff on student alcohol behaviour were found and all suggest that norms on alcohol as part of student life are important, Fig 2. Viewpoint I and VI suggest that students in general drink sensibly, and that alcohol consumption is not a driver when it comes to having fun with friends. In contrast to viewpoint I and VI, the staff members within viewpoint IX perceive that student drinking is problematic with students not considering the possible health effect of their behaviour. Six viewpoints (Viewpoint II—V and VII—VIII) sits in between these extremes, Fig 2. Viewpoint IV represents students who feel a social pressure to drink alcohol. Students in viewpoint II, III and VII have a perception that no one is or should feel pressured to drink alcohol if they do not want to. This despite the perception that drunk nights with friends do make some of the best memories for the time as a student. The students in viewpoint X think that alcohol plays such a dominant role, that students in general prioritize drinking over their studies. They themselves do not think that alcohol is needed to have a good party, which is a perception shared with the staff in viewpoint VIII. Students and staff members share the perception that students find drinking alcohol around friends as a good thing, and that alcohol consumption is part of student life and norm, Fig 2.

## Discussion

Five viewpoints for students and four for staff, were found in this study. All viewpoints share the perception that alcohol is part of university life, but complexity and contradictions within the viewpoints on the contribution alcohol makes to the university experience for students is apparent. The information found in this study provides important health promotion clues for developing different strategies to incentivize as many students as possible to drink less alcohol.

The general alcohol culture around the university setting also seems to play a role for the alcohol pattern of the students. Around fresher’s week and other social and mandatory activities at the university many students and staff members perceive that alcohol plays a dominant role. Some have a perception that it is fun and good thing, others feel pressure to participate, or participate because of social anxiety, as they fear missing out on something. This knowledge suggests intervening in a structural way to encourage staff and students to be active members of a healthy university environment might prove to be beneficial. For example, at University of Lund in Sweden throwing a party with alcohol on university premises is not permitted; staff and students are encouraged to keep the university premises free of alcohol [24–25]. Structural interventions like this could reduce the amount of alcohol being consumed at universities through developing and maintaining positive social norms amongst staff and students [26].

It is important to acknowledge the normative social influence on problematic alcohol intake when developing health promotion campaigns. Focusing on adjusting misperceptions and revealing an actual, healthier norm in the population as a whole or in certain sub-groups provides opportunity to develop positive norms and reduce risky alcohol consumption. Such a reduction will be to the advantage of both the individual and society [27]. The Social Norms Approach provides a theoretical framework for understanding the interaction between normative perceptions and normative behaviour [27]. Problematic alcohol use may largely be a dominant herd behaviour as students to a greater extent seem to mirror assumptions and perceptions of peer attitudes rather than the actual behaviour of peers [27]. Understanding such behavioural mechanisms are valuable to future health promotion interventions in university settings, where most students mainly consume alcohol in the company of others. By developing interventions based on the social norms approach most individuals will be encouraged to drink less alcohol by comparison to a realistic consumption pattern of peers, which may hold a positive public health outcome. Previous web-based interventions targeting alcohol behaviour exemplify this [28]. Theory based interventions targeting problematic alcohol use can potentially benefit from close attention to the interactions between the individual and the social and structural environment [12, 29–31], as reflected by their perceptions of alcohol e.g. documented in this study. Co-existing norms play a dominant role, shaping behavior [27–28].

### Study strength and limitations

By Q-sorting, the participants are in significant control of defining what is relevant within a phenomenon, and it is made possible to create distinct viewpoints [16]. To ensure this process, the developed concourse for the phenomenon needs to be applicable. The concourse used in this study was developed and pilot tested with students in an English university context. Without pilot testing in a local context it was transferred into a Danish university and applied to both staff and students. Potentially the concourse and statements development in the UK and the translations process of statements from English to Danish and the comments of the participants from Danish to English can have affected the quality, validity and reliability of this study. An over-representation of women (respectively 77% for students and 76% for staff) and medical students (55%) were found within study population. This was expected as in Aarhus, Denmark women study medicine, public health science and dentistry to a greater extent than men, but it does not correspond to the general gender distribution found within Danish universities [32].

Despite limitations, these findings serve as an important step in understanding the convergence and difference in perceptions among staff and students of the contribution alcohol makes to student life. In line with this study, the importance of using Q study typologies, extrapolating all perceptions and attitudes about the phenomenon, as a framework when developing public health interventions and policies to successfully prevent harmful alcohol consumption at universities, is also confirmed in a Q study of alcohol consumption in Irish university students [3]. Though students are the primary target population of alcohol interventions and campaigns in university settings, staff play a pivotal role in supporting such interventions through an articulation of concerns and risks related to alcohol and by supporting potential official alcohol policies of the universities. Students and staff in the health sciences are of particular concern in this regard, as their interactions, debates and educational focus with regard to alcohol is likely to affect the practice of the students in their future professional careers, where they will be guiding public norms and interventions on alcohol at different levels [8–9]. A future evaluation of the impact of interventions aiming to change the social norms around alcohol in university settings, like the structural change at University of Lund references above, would be highly relevant to better understand the relations between social norms, perceptions and attitudes to actual alcohol consumption patterns in a student population.

## Conclusion

Nine distinct viewpoints, five for students and four for staff, were found in this study. The viewpoints document different norms and perceptions around alcohol consumption as part of student life for university students. The varied norms and perceptions reflected in this study population is of relevance to international public health as it can help inform and better target the development of future health promotion intervention campaigns for changing behaviour and preventing harmful alcohol use in student populations.

## Supporting information

S1 Appendix Survey questions for students and staff. Copy of the survey questions for students and staff in the study.
(DOCX)
