# Supplementary material for: Student and staff perceptions of alcohol as part of student life in Denmark: A Q methodology study
Source: PLoS One. 2018 Oct 25;13(10):e0205923. doi: 10.1371/journal.pone.0205923 (PMC6201902; doi:10.1371/journal.pone.0205923)
Supplement: S1 Appendix — Copy of the survey questions for students and staff in the study. (DOCX) [file pone.0205923.s001.docx]

**S1 Appendix. Survey Questions for students and staff.**

**Copy of the survey questions for students in the study**

| Question number | Original text and questions in English* | Translation of the text and questions into Danish |
| --- | --- | --- |
| 1.01 | What is your age in years? | Hvor gammel er du? |
| 1.02 | What best describes your gender? | Hvad beskriver bedst dit køn? |
| 1.03 | In which department of Health are you a student at? | Hvilket institut under Health er du studerende ved? |
| 1.04 | What best describes your current student status? | Hvad beskriver bedst din aktuelle status som studerende? |
| 1.05 | Are you studying full-time or part-time? | Er du fuldtids- eller deltids- studerende? |
| 1.06 | Have you ever consumed a drink that contains alcohol? | Har du nogensinde drukket noget der indeholder alkohol? |
| 2.01 | How often do you have a drink containing alcohol | Hvor tit drikker du noget, der indeholder alkohol? |
| 2.02 | How many drinks containing alcohol do you have on a typical day when you are drinking? | Hvor mange genstande drikker du almindeligvis, når du drikker noget? |
| 2.03 | How often do you have six or more drinks on one occasion? | Hvor tit drikker du fem genstande eller flere ved samme lejlighed? |
| 2.04 | How often during the last 12 months have you found that you were not able to stop drinking once you had started? | Har du inden for det seneste år oplevet, at du ikke kunne stoppe når du først var begyndt at drikke? |
| 2.05 | How often during the last 12 months have you failed to do what was normally expected of you because of drinking? | Har du inden for det seneste år oplevet, at du ikke kunne gøre det du skulle, fordi du havde drukket? |
| 2.06 | How often during the last 12 months have you needed a first drink in the morning to get yourself going after a heavy drinking session? | Har du inden for det seneste år måtte have en lille én om morgenen efter, at du havde drukket meget dagen før? |
| 2.07 | How often during the last 12 months have you had a feeling of guilt or remorse after drinking? | Har du inden for det seneste år haft dårlig samvittighed eller fortrudt, efter du har drukket alkohol? |
| 2.08 | How often during the last 12 months have you been unable to remember what happened the night before because of your drinking? | Har du inden for det seneste år oplevet, at du ikke kunne huske hvad der skete aftenen før, fordi du havde drukket? |
| 2.09 | Not an item on the English AUDIT | Er du selv eller andre nogensinde kommet til skade ved en ulykke, fordi du havde drukket? |
| 2.10 | Not an item on the English AUDIT | Har nogen i familien, en ven, en læge eller andre været bekymret over dine alkoholvaner eller foreslået dig at sætte forbruget ned? |
| 3.01 | It is normal for students to use alcohol to relieve stress and worry | Det er normalt for studerende at anvende alkohol til at dulme stress og bekymringer |
| 3.02 | It can be expected that students will feel pressure from their peers to drink alcohol | Det kan forventes, at studerende føler sig presset af deres medstuderende til at drikke alkohol |
| 3.03 | Drinking alcohol at home before a night out is a normal part of student life | At drikke alkohol hjemmefra før man går i byen, er en normal del af studielivet |
| 3.04 | Being unable to remember parts of a night out due to drinking alcohol is an expected part of the student experience | At man ikke kan huske dele af aftenen i byen på grund af alkohol, er en forventelig del af at være studerende |
| 3.05 | Students are more interested in partying and drinking alcohol than studying | Studerende er mere interesserede i at feste og drikke alkohol end i at studere |
| 3.06 | After a student night out drinking alcohol it is accepted that there may be regrets the next day | Efter en aften ude med medstuderende, hvor man har drukket alkohol, er det acceptabelt, at der kan være ting, som man fortryder næste dag |
| 3.07 | Drinking a lot of alcohol is something students feel like they’re expected to do | At drikke en masse alkohol er noget, som studerende føler er forventet af dem |
| 3.08 | The way students behave when drunk is disgusting | Måden, som studerende opfører sig på, når de er fulde, er frastødende |
| 3.09 | Most students who prefer not to drink find it is easy to avoid alcohol | De fleste studerende, som foretrækker ikke at drikke, finder det nemt at undgå alkohol |
| 3.10 | Behaving like an idiot when drunk is seen as a normal student behaviour | At opføre sig som en idiot når man er fuld, opfattes som normal opførsel af en studerende |
| 3.11 | Students who are drunk don’t care about the consequences of their actions on others | Studerende, som er fulde, bekymrer sig ikke om konsekvenserne af deres opførsel overfor andre |
| 3.12 | A lot of student conversations revolve around alcohol and drinking | Mange samtaler mellem studerende drejer sig om alkohol og det at drikke |
| 3.13 | It is expected that academic work will be affected at some point due to the consequences of drinking alcohol | Det er forventeligt, at akademisk arbejde til en vis grad bliver påvirket af, at man har drukket alkohol |
| 3.14 | A good student party needs alcohol | En god fest kræver alkohol |
| 3.15 | Students that drink a lot of alcohol are often viewed as the most popular | Studerende, som drikker meget, er ofte de mest populære |
| 3.16 | Students view feeling unwell the next day after drinking too much alcohol as a sign of a good night out | Studerende, der har det dårligt dagen efter, at de har drukket, ser det som et tegn på, at de har haft en sjov aften i byen |
| 3.17 | Students that don’t drink alcohol are often viewed as boring | Studerende, som ikke drikker alkohol, bliver ofte set som kedelige |
| 3.18 | It is easier for students that drink alcohol to make friends | Det er nemmere for studerende, som drikker alkohol at få venner |
| 3.19 | Students live for today; they don’t think about the effects of alcohol on their health | Studerende lever i nuet og tænker ikke på alkoholens effekt på deres sundhed |
| 3.20 | Students enjoy spending a night at home with friends and not drinking, just as much as going out and drinking alcohol | Studerende nyder at bruge en aften hjemme med venner uden at drikke, lige så meget som at gå ud og drikke alkohol |
| 3.21 | It is expected that students can drink a lot of alcohol; it’s not acceptable for students to get drunk on just a few drinks | Det forventes, at studerende kan drikke meget alkohol, og det er ikke acceptabelt at blive fuld af blot nogle få genstande |
| 3.22 | Students get tired of social events that are organised around drinking alcohol | Studerende er trætte af social events, som er arrangeret omkring det at drikke alkohol |
| 3.23 | Students often drink alcohol so that they can fit in with other students | Studerende drikker ofte alkohol, så de passer ind sammen med andre studerende |
| 3.24 | Students like the fact that drinking alcohol lowers their inhibitions and enables them to do things they wouldn’t normally do | Studerende kan godt lide, at alkohol sænker deres hæmninger, så de har mulighed for at gøre ting, som de normalt ikke ville gøre |
| 3.25 | Most students drink sensibly or not at all | De fleste studerende drikker med fornuft eller overhovedet ikke 32. |
| 3.26 | The beginning of the academic year is typically all about getting drunk | Ved semesterstart handler alt typisk om at drikke sig fuld |
| 3.27 | Drinking alcohol is a strategy used to gain confidence by many students | Det at drikke alkohol bruges af mange studerende som en strategi til at øge deres selvtillid |
| 3.28 | Students think that getting drunk with friends is a good thing | Studerende synes, at det er en god ting at drikke sig fuld sammen med vennerne |
| 3.29 | An important part of the student experience is being free to drink alcohol | En vigtig del af oplevelsen som studerende er at være fri til at drikke alkohol |
| 3.30 | Being a student is the best time to drink alcohol because students have fewer responsibilities | Studietiden er det tidspunkt i livet, hvor man bedst kan indtage alkohol, fordi studerende har færre forpligtelser |
| 3.31 | Nights out with friends getting drunk provide some of the best memories of student life | Aftener med vennerne, hvor man drikker sig fuld, giver nogle af de bedste minder fra ens studietid |
| 3.32 | Students care more about being healthy these days and so the amount of alcohol they drink is reducing | Studerende gør mere for at være sunde i dag, så mængden af alkohol de drikker, er reduceret |
| 3.33 | Students think drinking alcohol blocks out negative emotions | Studerende mener at indtagelse af alkohol blokerer negative følelser. |
| 3.34 | Most students are good at knowing when to stop drinking alcohol so that they don’t get too drunk | De fleste studerende er gode til at fornemme, hvornår de skal stoppe med at drikke alkohol, så de ikke bliver for fulde |
| 3.35 | Students find it easy to admit to other students that they do not like drinking alcohol | Studerende finder det nemt at indrømme overfor andre studerende, at de ikke kan lide at drikke alkohol |
| 3.36 | Students are thoughtful about when to drink alcohol, taking academic obligations such as essays or exams into account | Studerende tænker over, hvornår de drikker alkohol, og tager højde for akademiske forpligtelser såsom opgaver eller eksamener |
| 3.37 | Games that involve drinking alcohol are a valued part of the student drinking experience | Spil som involverer alkohol er en betydningsfuld del af de studerendes alkoholoplevelse |
| 3.38 | For students who work hard, a night out drinking alcohol and getting drunk is well-deserved | Studerende som arbejder hårdt fortjener en aften i byen, hvor de kan drikke sig fulde |
| 3.39 | Students spend too much money on alcohol rather than items like food or academic books | Studerende burger for mange penge på alkohol set I forhold til ting som mad og akademiske bøger |
| 3.40 | It is normal to see students finishing their drinks fast and in one go so they get more effect from the alcohol | Det er normalt at se studerende indtage drinks hurtigt og på en gang for at opnå større effekt af alkoholen |

* The English survey questions have previously been published (Yule R. 2014).

Question number: 1.01-1.06 = Demographic questions

Question number: 2.01-2.10 = Alcohol Use Disorders Identification Test (AUDIT)

Question number: 3.01-3.40 = Questions for the Q-study

**Copy of the survey questions for staff in the study**

| Question number | Original text and questions in English* | Translation of the text and questions into Danish |
| --- | --- | --- |
| 1.01 | What is your age in years? | Hvor gammel er du? |
| 1.02 | What best describes your gender? | Hvad beskriver bedst dit køn? |
| 1.03 | In which department of Health are you a student at? | Hvilket institut under Health er du studerende ved? |
| 1.04 | Which department at Health are you employed at? | Hvilket institut under Health er du ansat ved? |
| 1.05 | What is the best description of your employment at the Institute/University of Aarhus? | Hvad beskriver bedst din ansættelse på Institut/Aarhus Universitet? |
| 1.06 | What type of employment do you have? | Hvilken type ansættelse har du? |
| 1.07 | What is the best description of your role as VIP / TAP? | Hvad beskriver bedst din rolle som VIP / TAP? |
| 1.08 | How must contact do you have with students? | Hvor meget kontakt har du til studerende? |
| 1.09 | Have you ever consumed a drink that contains alcohol? | Har du nogensinde drukket noget der indeholder alkohol? |
| 2.01 | How often do you have a drink containing alcohol | Hvor tit drikker du noget, der indeholder alkohol? |
| 2.02 | How many drinks containing alcohol do you have on a typical day when you are drinking? | Hvor mange genstande drikker du almindeligvis, når du drikker noget? |
| 2.03 | How often do you have six or more drinks on one occasion? | Hvor tit drikker du fem genstande eller flere ved samme lejlighed? |
| 2.04 | How often during the last 12 months have you found that you were not able to stop drinking once you had started? | Har du inden for det seneste år oplevet, at du ikke kunne stoppe når du først var begyndt at drikke? |
| 2.05 | How often during the last 12 months have you failed to do what was normally expected of you because of drinking? | Har du inden for det seneste år oplevet, at du ikke kunne gøre det du skulle, fordi du havde drukket? |
| 2.06 | How often during the last 12 months have you needed a first drink in the morning to get yourself going after a heavy drinking session? | Har du inden for det seneste år måtte have en lille én om morgenen efter, at du havde drukket meget dagen før? |
| 2.07 | How often during the last 12 months have you had a feeling of guilt or remorse after drinking? | Har du inden for det seneste år haft dårlig samvittighed eller fortrudt, efter du har drukket alkohol? |
| 2.08 | How often during the last 12 months have you been unable to remember what happened the night before because of your drinking? | Har du inden for det seneste år oplevet, at du ikke kunne huske hvad der skete aftenen før, fordi du havde drukket? |
| 2.09 | Not an item on the English AUDIT | Er du selv eller andre nogensinde kommet til skade ved en ulykke, fordi du havde drukket? |
| 2.10 | Not an item on the English AUDIT | Har nogen i familien, en ven, en læge eller andre været bekymret over dine alkoholvaner eller foreslået dig at sætte forbruget ned? |
| 3.01 | It is normal for students to use alcohol to relieve stress and worry | Det er normalt for studerende at anvende alkohol til at dulme stress og bekymringer |
| 3.02 | It can be expected that students will feel pressure from their peers to drink alcohol | Det kan forventes, at studerende føler sig presset af deres medstuderende til at drikke alkohol |
| 3.03 | Drinking alcohol at home before a night out is a normal part of student life | At drikke alkohol hjemmefra før man går i byen, er en normal del af studielivet |
| 3.04 | Being unable to remember parts of a night out due to drinking alcohol is an expected part of the student experience | At man ikke kan huske dele af aftenen i byen på grund af alkohol, er en forventelig del af at være studerende |
| 3.05 | Students are more interested in partying and drinking alcohol than studying | Studerende er mere interesserede i at feste og drikke alkohol end i at studere |
| 3.06 | After a student night out drinking alcohol it is accepted that there may be regrets the next day | Efter en aften ude med medstuderende, hvor man har drukket alkohol, er det acceptabelt, at der kan være ting, som man fortryder næste dag |
| 3.07 | Drinking a lot of alcohol is something students feel like they’re expected to do | At drikke en masse alkohol er noget, som studerende føler er forventet af dem |
| 3.08 | The way students behave when drunk is disgusting | Måden, som studerende opfører sig på, når de er fulde, er frastødende |
| 3.09 | Most students who prefer not to drink find it is easy to avoid alcohol | De fleste studerende, som foretrækker ikke at drikke, finder det nemt at undgå alkohol |
| 3.10 | Behaving like an idiot when drunk is seen as a normal student behaviour | At opføre sig som en idiot når man er fuld, opfattes som normal opførsel af en studerende |
| 3.11 | Students who are drunk don’t care about the consequences of their actions on others | Studerende, som er fulde, bekymrer sig ikke om konsekvenserne af deres opførsel overfor andre |
| 3.12 | A lot of student conversations revolve around alcohol and drinking | Mange samtaler mellem studerende drejer sig om alkohol og det at drikke |
| 3.13 | It is expected that academic work will be affected at some point due to the consequences of drinking alcohol | Det er forventeligt, at akademisk arbejde til en vis grad bliver påvirket af, at man har drukket alkohol |
| 3.14 | A good student party needs alcohol | En god fest kræver alkohol |
| 3.15 | Students that drink a lot of alcohol are often viewed as the most popular | Studerende, som drikker meget, er ofte de mest populære |
| 3.16 | Students view feeling unwell the next day after drinking too much alcohol as a sign of a good night out | Studerende, der har det dårligt dagen efter, at de har drukket, ser det som et tegn på, at de har haft en sjov aften i byen |
| 3.17 | Students that don’t drink alcohol are often viewed as boring | Studerende, som ikke drikker alkohol, bliver ofte set som kedelige |
| 3.18 | It is easier for students that drink alcohol to make friends | Det er nemmere for studerende, som drikker alkohol at få venner |
| 3.19 | Students live for today; they don’t think about the effects of alcohol on their health | Studerende lever i nuet og tænker ikke på alkoholens effekt på deres sundhed |
| 3.20 | Students enjoy spending a night at home with friends and not drinking, just as much as going out and drinking alcohol | Studerende nyder at bruge en aften hjemme med venner uden at drikke, lige så meget som at gå ud og drikke alkohol |
| 3.21 | It is expected that students can drink a lot of alcohol; it’s not acceptable for students to get drunk on just a few drinks | Det forventes, at studerende kan drikke meget alkohol, og det er ikke acceptabelt at blive fuld af blot nogle få genstande |
| 3.22 | Students get tired of social events that are organised around drinking alcohol | Studerende er trætte af social events, som er arrangeret omkring det at drikke alkohol |
| 3.23 | Students often drink alcohol so that they can fit in with other students | Studerende drikker ofte alkohol, så de passer ind sammen med andre studerende |
| 3.24 | Students like the fact that drinking alcohol lowers their inhibitions and enables them to do things they wouldn’t normally do | Studerende kan godt lide, at alkohol sænker deres hæmninger, så de har mulighed for at gøre ting, som de normalt ikke ville gøre |
| 3.25 | Most students drink sensibly or not at all | De fleste studerende drikker med fornuft eller overhovedet ikke 32. |
| 3.26 | The beginning of the academic year is typically all about getting drunk | Ved semesterstart handler alt typisk om at drikke sig fuld |
| 3.27 | Drinking alcohol is a strategy used to gain confidence by many students | Det at drikke alkohol bruges af mange studerende som en strategi til at øge deres selvtillid |
| 3.28 | Students think that getting drunk with friends is a good thing | Studerende synes, at det er en god ting at drikke sig fuld sammen med vennerne |
| 3.29 | An important part of the student experience is being free to drink alcohol | En vigtig del af oplevelsen som studerende er at være fri til at drikke alkohol |
| 3.30 | Being a student is the best time to drink alcohol because students have fewer responsibilities | Studietiden er det tidspunkt i livet, hvor man bedst kan indtage alkohol, fordi studerende har færre forpligtelser |
| 3.31 | Nights out with friends getting drunk provide some of the best memories of student life | Aftener med vennerne, hvor man drikker sig fuld, giver nogle af de bedste minder fra ens studietid |
| 3.32 | Students care more about being healthy these days and so the amount of alcohol they drink is reducing | Studerende gør mere for at være sunde i dag, så mængden af alkohol de drikker, er reduceret |
| 3.33 | Students think drinking alcohol blocks out negative emotions | Studerende mener at indtagelse af alkohol blokerer negative følelser. |
| 3.34 | Most students are good at knowing when to stop drinking alcohol so that they don’t get too drunk | De fleste studerende er gode til at fornemme, hvornår de skal stoppe med at drikke alkohol, så de ikke bliver for fulde |
| 3.35 | Students find it easy to admit to other students that they do not like drinking alcohol | Studerende finder det nemt at indrømme overfor andre studerende, at de ikke kan lide at drikke alkohol |
| 3.36 | Students are thoughtful about when to drink alcohol, taking academic obligations such as essays or exams into account | Studerende tænker over, hvornår de drikker alkohol, og tager højde for akademiske forpligtelser såsom opgaver eller eksamener |
| 3.37 | Games that involve drinking alcohol are a valued part of the student drinking experience | Spil som involverer alkohol er en betydningsfuld del af de studerendes alkoholoplevelse |
| 3.38 | For students who work hard, a night out drinking alcohol and getting drunk is well-deserved | Studerende som arbejder hårdt fortjener en aften i byen, hvor de kan drikke sig fulde |
| 3.39 | Students spend too much money on alcohol rather than items like food or academic books | Studerende burger for mange penge på alkohol set I forhold til ting som mad og akademiske bøger |
| 3.40 | It is normal to see students finishing their drinks fast and in one go so they get more effect from the alcohol | Det er normalt at se studerende indtage drinks hurtigt og på en gang for at opnå større effekt af alkoholen |

* The English survey questions have previously been published (Yule R. 2014).

Question number: 1.01-1.06 = Demographic questions

Question number: 2.01-2.10 = Alcohol Use Disorders Identification Test (AUDIT)

Question number: 3.01-3.40 = Questions for the Q-study
